# Osteostatin Inhibits Collagen-Induced Arthritis by Regulation of Immune Activation, Pro-Inflammatory Cytokines, and Osteoclastogenesis

**DOI:** 10.3390/ijms20163845

**Published:** 2019-08-07

**Authors:** Josep Nácher-Juan, María Carmen Terencio, María José Alcaraz, María Luisa Ferrándiz

**Affiliations:** Instituto Interuniversitario de Investigación de Reconocimiento Molecular y Desarrollo Tecnológico (IDM), Universitat Politècnica de València, Universitat de València, Av. Vicent A. Estellés s/n, 46100 Burjasot, Valencia, Spain

**Keywords:** osteostatin, arthritis, inflammation, immune response, cartilage destruction, bone erosion

## Abstract

In chronic inflammatory joint diseases, such as rheumatoid arthritis, there is an important bone loss. Parathyroid hormone-related protein (PTHrP) and related peptides have shown osteoinductive properties in bone regeneration models, but there are no data on inflammatory joint destruction. We have investigated whether the PTHrP (107-111) C-terminal peptide (osteostatin) could control the development of collagen-induced arthritis in mice. Administration of osteostatin (80 or 120 μg/kg s.c.) after the onset of disease decreased the severity of arthritis as well as cartilage and bone degradation. This peptide reduced serum IgG2a levels as well as T cell activation, with the downregulation of RORγt+CD4+ T cells and upregulation of FoxP3+CD8+ T cells in lymph nodes. The levels of key cytokines, such as interleukin(IL)-1β, IL-2, IL-6, IL-17, and tumor necrosis factor-α in mice paws were decreased by osteostatin treatment, whereas IL-10 was enhanced. Bone protection was related to reductions in receptor activator of nuclear factor-κB ligand, Dickkopf-related protein 1, and joint osteoclast area. Osteostatin improves arthritis and controls bone loss by inhibiting immune activation, pro-inflammatory cytokines, and osteoclastogenesis. Our results support the interest of osteostatin for the treatment of inflammatory joint conditions.

## 1. Introduction

Parathyroid hormone (PTH) and parathyroid hormone-related protein (PTHrP) show structural homology at the N-terminal region, which determines the interaction with the common PTH type 1 receptor. The bone anabolic properties of different PTHrP-derived peptides have been demonstrated in vitro and in vivo. In particular, the Food and Drug Administration have approved abaloparatide, which is an analogue that is based on the N-terminal 1-34 sequence of human PTHrP, for the treatment of postmenopausal women with osteoporosis at high risk for fracture. Several studies have demonstrated that both the N-terminal fragment and the PTH-unrelated C-terminal domain of PTHrP can enhance osteoblast proliferation and differentiation [1,2] and it can confer osteoinductive effects to implants in animal models of bone repair [3]. In addition, some C-terminal peptides have been found to reduce osteoclast activity in vitro, suggesting that the C-terminal PTHrP may act as a paracrine regulator of bone metabolism [4].

Rheumatoid arthritis (RA) is the most common chronic inflammatory joint disease. Autoimmunity responses precede joint inflammation and cellular activation to release a wide range of pro-inflammatory and catabolic mediators inducing the chronic inflammatory state and articular damage [5]. RA is characterized by an important alteration in bone homeostasis with an imbalance between bone resorption and formation [6]. Therefore, localized bone resorption and generalized bone loss are both associated to the progression of disease [7]. Bone damage arises from complex interactions that determine osteoclast precursor maturation that is mainly mediated by receptor activator of nuclear factor-κB ligand (RANKL), macrophage colony-stimulating factor and tumor necrosis factor-α (TNFα) pathways [5].

High levels of PTHrP in synovial fluid from RA patients are thought to participate in the regulation of different articular cells, such as subchondral bone osteoblasts, lymphocytes, or chondrocytes [8]. The production of PTHrP has been related to chondrogenesis and the inhibition of hypertrophic development of mesenchymal stem cells [9]. Indeed, PTHrP delays chondrocyte differentiation and it is involved in cartilage maturation [10]. Nevertheless, some report has suggested a pathogenic role for PTHrP that is related to synovial cell proliferation or osteoclast induction in arthritic articular tissues [11].

We have recently shown that PTHrP peptides are able to control senescence and inflammation in osteoarthritic osteoblasts. These properties were mainly associated to the C-terminal moiety of these agents [12]. No studies have yet addressed if these peptides could control joint inflammatory conditions. Therefore, we have explored whether PTHrP (107-111) (osteostatin), which includes the -Thr-Arg-Ser-Ala-Trp- sequence of the PTHrP C terminal fragment, exerts inhibitory effects on inflammation and joint degradation in the collagen-induced arthritis (CIA). This model exhibits morphological features that are similar to RA, including synovitis and erosion of cartilage and bone [13].

## 2. Results

### 2.1. Effects of Osteostatin on the Progression of Arthritis

**Figure 1:**
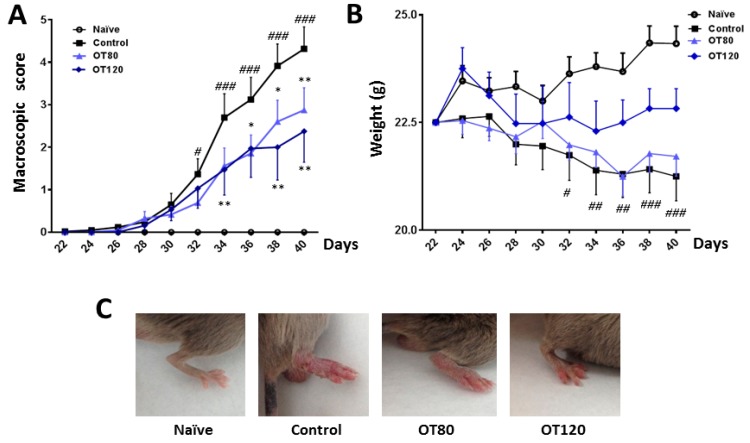
(**A**) Time course of arthritis macroscopic score. For each animal, a total score value was calculated after the second immunization on day 21. (**B**) Time course of body weight. (**C**) Representative images of mice hindpaw on day 40. Data are presented as mean ± SEM (*n* = 8 mice per group). # *p* < 0.05, ## *p* < 0.01, ### *p* < 0.001 versus naïve group; * *p* < 0.05, ** *p* < 0.01 versus control group. Two-way ANOVA (Bonferroni post-test).

As shown in Figure 1A, the severity of arthritis increased over time, until the end of the experiment. Mice that were treated with osteostatin (80 μg/kg or 120 μg/kg) showed a sustained reduction in this score, which was significant from days 34 to 40.

Figure 1C shows representative images of these experimental groups. The progression of the arthritic process resulted in weight loss when compared with naïve animals, while the treatment with the highest dose of osteostatin tended to normalize this parameter (Figure 1B).

### 2.2. Joint Histological Analysis

**Figure 2:**
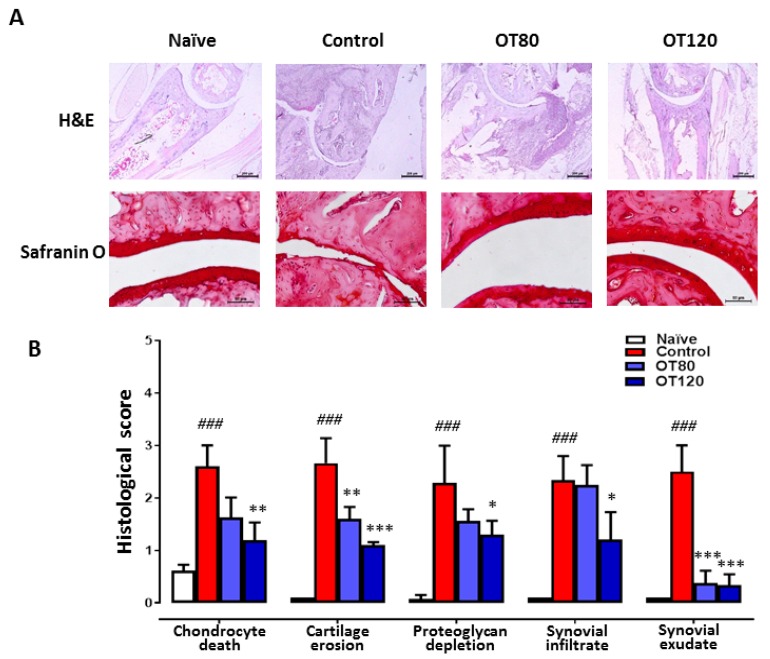
Histological analysis of ankle joints at day 40. (**A**) Haematoxylin and eosin (H&E) and safranin O stained sections (40× and 200×, respectively). Bar = 200 μm (H&E) and 50 μm (safranin O). (**B**) The histological scores are presented as mean ± S.D. (*n* = 5) and were analyzed using one-way ANOVA with Tukey’s post-test. ### *p* < 0.001 versus naïve group; * *p* < 0.05, ** *p* < 0.01, *** *p* < 0.001 versus control group.

Cartilage pathology and joint inflammation were assessed by joint histological analysis of ankle sections that were stained with haematoxylin/eosin or safranin O. Representative sections are shown in Figure 2A and the histological score in Figure 2B. Histopathological examination of ankle joints of arthritic control mice showed cartilage damage with chondrocyte death and proteoglycan depletion, besides synovial infiltrate and exudate. Treatment with both doses of osteostatin led to reductions in chondrocyte death, cartilage erosion, proteoglycan depletion, and synovial exudate, while a reduction in synovial infiltrate was observed at the highest dose.

### 2.3. Effects on Local Cytokine Levels and Myeloperoxidase Activity

**Figure 3:**
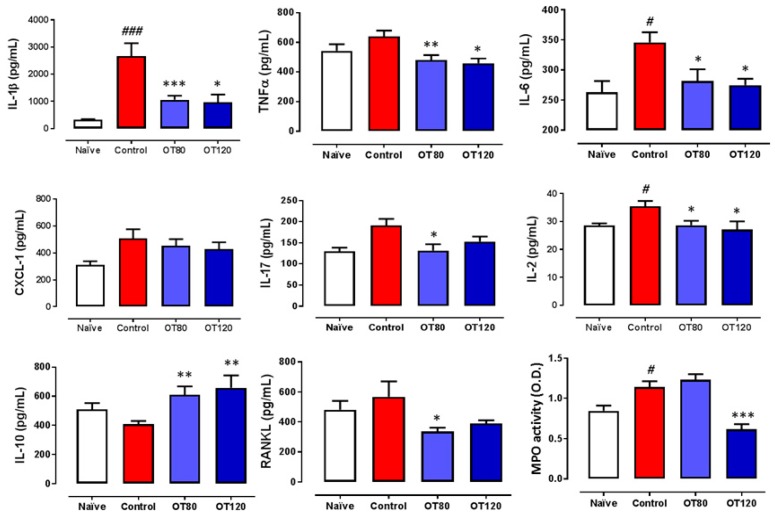
Levels of mediators in paw homogenates. Cytokines were measured by ELISA. Myeloperoxidase (MPO) activity was determined by spectrophotometry O.D. (optical density, 450 nm). Data presented as mean ± SEM. # *p* < 0.05, ### *p* < 0.001 versus naïve group; * *p* < 0.05, ** *p* < 0.01, *** *p* < 0.001 versus control group. One-way ANOVA (Tukey’s post-test with *n* = 8).

Next, we investigated whether osteostatin could regulate local cytokines. For this purpose, we measured by ELISA the levels of key cytokines present in paw homogenates at the end of the experiment. We found a significant reduction of interleukin(IL)-1β, TNFα, IL-6, and IL-2 by both doses of osteostatin, whereas no significant effect was observed on the chemotactic factor CXCL-1 (Figure 3). In addition, the levels of IL-17 and RANKL (osteoclast-differentiation factor) were decreased by both doses of osteostatin, although the results reached statistical significance for the 80 μg/kg dose. Notably, the levels of the anti-inflammatory cytokine IL-10 were significantly enhanced by both doses of osteostatin. Myeloperoxidase (MPO) activity was measured in paw homogenates. As shown in Figure 3, MPO was increased by arthritis induction, and this effect was significantly reduced by the highest dose of osteostatin.

### 2.4. Effects on Serum IgG2a and Bone Metabolism Biomarkers

**Figure 4:**
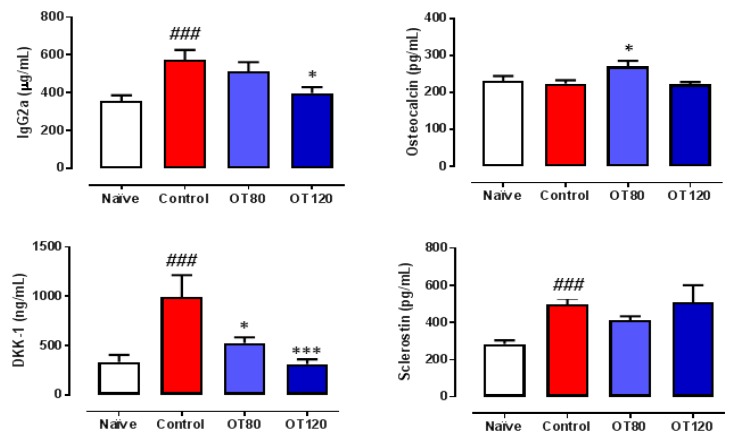
Serum levels of IgG2a and bone metabolism biomarkers Dickkopf-related protein 1 (DKK-1), sclerostin, and osteocalcin. IgG2a levels were measured by ELISA and bone metabolism biomarkers by multiplexing. Data are presented as mean ± SEM. One-way ANOVA with Tukey’s post-test. ### *p* < 0.001, versus naïve group; * *p* < 0.05, *** *p* < 0.001 versus control group (*n* = 8).

As the severity of arthritis in this experimental model is positively correlated with the IgG autoantibody response to collagen II [14] and the presence of the IgG2a isotype [15], we wanted to investigate whether osteostatin can modify the serum levels of IgG2a. ELISA analyzed serum samples that were taken at the end of the study. Figure 4 shows that the IgG2a levels were significantly increased in arthritic control mice, while osteostatin treatment reduced them, although statistical significance was only reached for the 120 μg/kg dose. In addition, several biomarkers of bone metabolism were measured in serum. The wingless-related integration site (Wnt) signaling pathways play an essential role in regulating bone development and homeostasis of joints and the skeleton mass [16]. Arthritis induction did not modify osteocalcin (a biomarker of mature osteoblasts) levels, but enhanced the Wnt regulatory molecules sclerostin and Dickkopf-related protein 1 (DKK-1) (Figure 4). Our results indicate that DKK-1 levels were dose-dependently inhibited by osteostatin, whereas osteocalcin and sclerostin were not significantly modified.

### 2.5. Lymph Node T Cell Proliferation and Release of Cytokines

**Figure 5:**
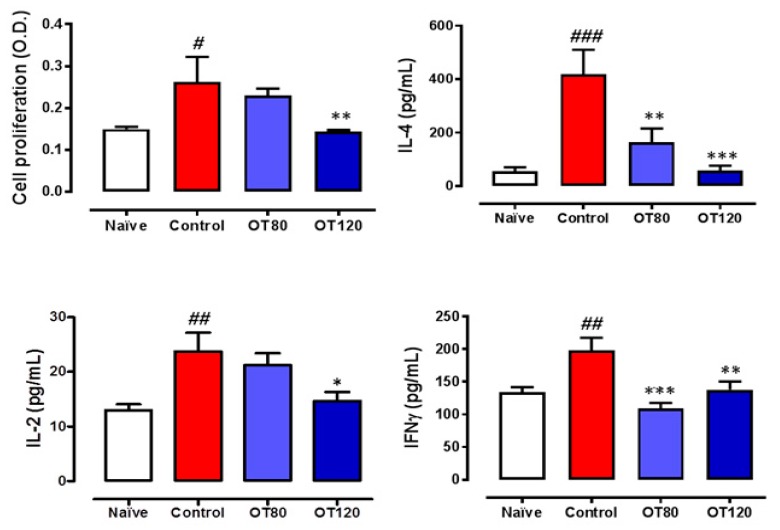
T cell proliferation assay and release of cytokines. Lymph node T cell proliferation was measured by bromdeoxiuridine incorporation. O.D. (optical density, 405 nm). ELISA measured levels of T cell differentiation cytokines in the conditioned media of the cell proliferation assay. Data are presented as mean ± SEM (*n* = 5), one-way ANOVA with Tukey’s post-test. # *p* < 0.05, ## *p* < 0.01, ### *p* < 0.001 versus naïve group; * *p* < 0.05, ** *p* < 0.01, *** *p* < 0.001 versus control group.

We next determined the effects of osteostatin on T cells that were present in lymph nodes. Figure 5 shows that T cell proliferation was stimulated in arthritic control mice versus naïve group. A lower proliferation was observed in osteostatin-treated animals, with a significant effect for the highest dose, which reduced proliferation to levels similar to those found in naïve animals. In addition, T cells from arthritic control mice released higher levels of the cytokines IL-2, IL-4, and interferon γ (IFNγ) as compared with non-arthritic animals. The treatment with osteostatin reduced the release of these cytokines with significant effects for IL-2 at the highest dose and for IL-4 and IFNγ at both doses.

**Figure 6:**
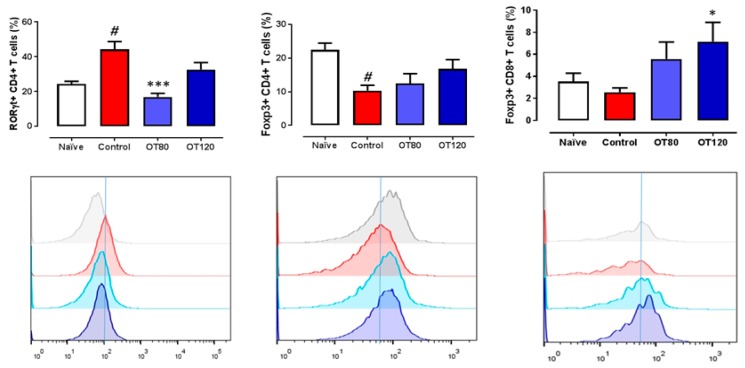
Flow cytometry analysis of lymph node T cell populations. FoxP3+ and RORγt+ T cell populations are represented. Data are mean ± SEM (*n* = 5), one-way ANOVA with Tukey’s post-test. # *p* < 0.05 versus naïve group; * *p* < 0.05, *** *p* < 0.001 versus control group.

The analysis of T cell populations revealed that arthritis induction increased the number of RORγt+CD4+ T cells, whereas Foxp3+CD4+ T cells and to a lower extent Foxp3+CD8+ T cells were reduced in arthritic controls when compared with naïve mice (Figure 6).

Osteostatin reverted the effect of CIA on RORγt+CD4+ T cells and tended to enhance Foxp3+CD4+ T cells. In addition, osteostatin increased the number of Foxp3+CD8+T cells, with statistically significant results for the 120 μg/kg dose.

### 2.6. Bone Degradation

**Figure 7:**
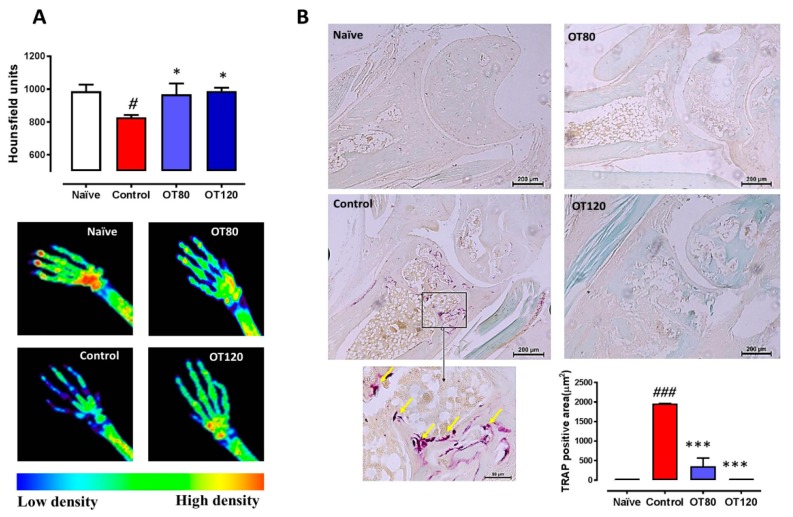
Bone density and osteoclast area analysis. (**A**) X-rays analysis of both front and hind limbs. Bone density was measured by analyzing a ROI (region of interest) around the wrist and ankle joints. Values are presented in Hounsfield units. Representative images of hind paws with color scale: red, high bone density; green/yellow, middle density; blue, low density (*n*= 5). (**B**) Tartrate-resistant acid phosphatase (TRAP) staining of mice ankle sections. The purple staining represent the TRAP positive area associated to osteoclasts activity. Bar = 200 μm and 50 μm. Data are presented as mean ± SEM (*n* = 5). One-way ANOVA with Tukey’s post-test. # *p* < 0.05, ### *p* < 0.001 versus naïve group; * *p* < 0.05, *** *p* < 0.001 versus control group.

After identifying histological improvements in ankle joint by osteostatin treatment, we checked whether this compound could modify arthritic bone alterations. Analysis of X-rays taken from the mice paws revealed that osteostatin treatment reduced the severity of radiographic changes. Figure 7A shows the quantification of bone density (front and hind paws) in Hounsfield units and representative images of hind paws. Arthritis induction significantly reduced bone density while osteostatin normalized these values. The differentiation of osteoclasts is a key process driving major erosive lesions in RA [17]. Osteoclast area was assessed by tartrate-resistant acid phosphatase (TRAP) staining of mice ankle sections. Arthritic control mice showed a significant enhancement of TRAP staining when compared with non-arthritic animals (Figure 7B). Of note, osteostatin dose-dependently reduced TRAP staining, which showed values that were similar to those of naïve mice in animals that were treated with the 120 μg/kg dose.

## 3. Discussion

In the present study, we show that osteostatin treatment ameliorates the severity of experimental arthritis with efficacy on clinical signs and structural alterations of CIA. Mice injected with collagen II develop arthritis that is similar in many respects to human RA characterized by a progressive breakdown of articular cartilage and bone erosion. Histological analysis revealed a significant improvement in inflammation, proteoglycan loss, and cartilage damage by osteostatin administration as compared with arthritic control mice. Pro-inflammatory cytokines are abundantly expressed in the arthritic joints of CIA mice as in human RA. In particular, TNFα plays a relevant role in the integration of the arthritic response, as demonstrated by the therapeutic efficacy of different strategies aimed at blocking this cytokine [18]. Our results indicate that osteostatin treatment reduces cell influx into the synovium, as well as the number of cells that were present in the joint cavity and the levels of key pro-inflammatory cytokines, such as TNFα, IL-1β, and IL-6. High levels of IL-10 are present in synovial tissues of RA patients, where it may have an immunoregulatory function [19,20]. Interestingly, osteostatin treatment significantly enhanced the local production of IL-10, which may be contribute to the control of disease severity as the deficiency of this cytokine exacerbates the CIA response [21].

Genetically linked autoimmunity to collagen II leads to the induction of arthritis and it may be determinant for the chronicity of the process and joint cartilage destruction [22,23]. We found that osteostatin is able to downregulate the production of anti-collagen II IgG2a, an autoantibody subclass that appears to be of particular importance in the CIA model [15]. The adaptive immune response and additional mechanisms play a role in the progression of CIA [24]. It has been suggested that collagen II-specific T cells act during the effector phase of arthritis, leading to perpetuation and exacerbation of disease [25]. CD4+ T cells differentiate into several subsets that are involved in the arthritic process [26]. In particular, IL-17-producing Th17 cells appear to play a predominant role in autoimmune arthritis [27]. Our data indicate that osteostatin affects T cell immunity in the CIA model. Therefore, this peptide may downregulate the activity of Th17 cells, as it reduced the number of RORγt+CD4+ T cells in the lymph node and the levels of IL-17 in the joints [28]. In addition, osteostatin counteracted the enhanced production of IL-2, IFNγ (Th1 activity), and IL-4 (Th2 activity) induced by the arthritic process. Accumulating evidence has revealed a key role of IL-17 in the production of pro-inflammatory cytokines [29] and the potentiation of their effects for cartilage degradation [30]. Furthermore, IL-17 is an important inducer of RANKL expression stimulating osteoclastogenesis and bone erosion in arthritis [31]. These results suggest that osteostatin might inhibit T cell activation and the subsequent generation of cytokines that play key roles in the pathogenesis of arthritis and joint degradation.

On the other hand, it has become evident that CD4+ regulatory T cells (Tregs) are involved in the control of clinical symptoms of CIA, cytokine production, effector T cell activity [32], and osteoclastogenesis [33,34]. As a result, the enhancement of Treg activity may be beneficial for the treatment of inflammation-induced bone loss [35]. In addition to CD4+ Tregs, CD8+ Tregs possess important immunosuppressive functions [36] and regulate Th17-mediated autoimmune diseases [37]. Interestingly, CD8+ Tregs are present in synovium from RA patients and they have the ability to decrease the activity of lesional T cells as well as the production of proinflammatory cytokines, suggesting a role in synovitis downregulation and potential therapeutic applications (reviewed in [38]). Thus, induced human CD8+Tregs have been shown to alleviate the severity of CIA and inhibit the mRNA expression of IL-17A and RANKL in mouse paw [39]. We found that osteostatin enhances CD8+Treg cells, which may contribute to the downregulation of the immune response to collagen II and the development of arthritis.

The control of bone destruction is a relevant objective in RA treatment. The erosion of subchondral and cortical bone is common in areas of synovial hyperplasia. Besides, bone destruction is associated to the presence of osteoclasts and RANKL expression [40]. X-rays analysis of mouse limbs showed that osteostatin significantly counteracted the bone density loss that is induced by the arthritic process. High levels of pro-inflammatory cytokines and mainly TNFα produced in inflammatory arthritis alter the resorption/formation cycle necessary for bone homeostasis. TNFα may contribute to the inhibition of osteoblast maturation and function acting directly on these cells [41] or through the modulation of the Wnt pathway. In fact, TNFα is a key inducer of the Wnt inhibitor DKK-1 in mouse inflammatory arthritis and in human RA [42]. In addition to the upregulation of RANKL expression by pro-inflammatory cytokines [43], it is known that TNFα cooperates with RANKL for osteoclast differentiation [41]. Furthermore, TNFα can stimulate osteoclast formation by RANKL-independent mechanisms [44]. The bone protective effect of osteostatin that is reported here was supported by reductions in osteoclast numbers and the inhibition of pro-inflammatory cytokines, such as TNFα and RANKL, suggesting that osteostatin is able to inhibit osteoclastogenesis in vivo, a critical process in the pathogenesis of joint damage. These results extend previous findings on the inhibitory effects of PTHrP C-terminal peptides on osteoclast function in vitro [4].

The Wnt pathway plays an important role in joint remodeling. It is generally accepted that members of the Wnt family are involved in the production of pro-inflammatory cytokines or catabolic enzymes in RA [45]. The inhibition of Wnt signaling may be responsible for the suppression of normal osteoblast function at sites of bone erosion in RA [42,46]. It is also known that DKK1 enhances RANKL expression and facilitates osteoclastogenesis [47]. Interestingly, the DKK1 levels are elevated in sera from early RA patients and correlate with disease activity [48]. Thus, recent studies have reported the reduction in DKK1 serum levels in RA patients in remission. In contrast, sclerostin levels do not correlate with disease activity and they do not change during remission [49]. In experimental arthritis, neutralization of DKK-1 with an antibody inhibited bone erosion without affecting inflammation [42], whereas the neutralization of sclerostin improved systemic bone loss, but did not affect disease severity or focal bone erosions in the CIA model [50]. However, this last approach worsened clinical outcome in models of TNFα-dependent inflammation, as sclerostin has an inhibitory effect on TNFα-induced p38 activation [51]. In line with these findings, we have observed a strong inhibitory effect of osteostatin on the serum levels of DKK-1 without a significant modification of sclerostin levels in CIA mice which suggests that DKK-1 downregulation may play a role in the inhibition of arthritic bone loss by osteostatin treatment.

Our data have shown the anti-arthritic properties of the oligopeptide osteostatin. It is interesting to note that oligopeptides may have some advantages for drug development when compared with higher molecular weight products, such as convenient synthesis and low immunogenicity, as well as favorable pharmacokinetics. However, some studies have reported that oligopeptides show an acceptable in vivo stability and integrity, good tissue penetration to exert their effects and a low toxicity. Additionally, they have gained wide attention for drug delivery systems and different modifications can be easily applied to improve their pharmacological profile [52]. All of these properties are relevant regarding potential therapeutic applications.

In conclusion, our results demonstrate the protective effects of osteostatin in a model of RA. This agent downregulated the immune and inflammatory responses resulting in reduced cartilage and bone destruction. The bone protective effects of osteostatin can be dependent on the inhibition of osteoclastogenesis, which may be mediated by the downregulation of key cytokines and DKK-1. Therefore, C-terminal PTHrP peptides provide an interesting approach for developing novel therapeutic opportunities for inflammatory joint conditions.

## 4. Materials and Methods

### 4.1. Animals

Male DBA/1 mice (Janvier, Le Genest-Saint-Isle, France) between 10 and 12 weeks of age (18–20 g) were used for all experiments. All the mice were housed in plastic cages (four per cage) with wood chips for bedding in a quiet room under controlled lighting (12 h day/night cycle) and temperature (22 ± 1 °C). Standard diet and water were provided ad libitum. All of the experiments were performed in accordance with European regulations for the handling and use of laboratory animals (Directive 2010/63/EU and Spanish R. D. 53/2013). The Institutional Animal Care and Use Committee (Comité de Etica de Experimentación Animal de la Universidad de Valencia, Spain) approved the protocols (number 2016/VSC/PEA/00053, 11 June 2016). All of the studies are reported in accordance with the ARRIVE guidelines for reporting experiments involving animals [53].

### 4.2. Induction of Arthritis

Arthritis was induced, as previously described [54]. Bovine type II collagen (2 mg/mL) (Chondrex Inc. Redmond, WA, USA) was emulsified in equal volumes of Freund’s complete adjuvant (Thermofisher Scientific Inc. Waltham, MA, USA). On day 0, the mice were immunized by subcutaneous (s.c.) injection of the emulsion (100 μL) at the base of the tail. On day 21, animals received an intraperitoneal booster injection of collagen II (2 mg/mL, 100 μL) that was dissolved in phosphate buffered saline (PBS).

### 4.3. Experimental Groups and Treatment

Mice were randomly assigned to experimental groups: naïve group, which was not immunized (*n* = 8); control group, which was immunized but not treated (*n* = 8); OT80, which was immunized and treated with 80 μg/kg per day of osteostatin (Bachem AG, Bubendorf, Switzerland) in physiological saline (*n* = 8); and, OT120, which was immunized and treated with 120 μg/kg per day of osteostatin in physiological saline (*n* = 8). Doses, route, and frequency of administration were selected in preliminary experiments. The treated mice received 100 μL per day (s.c.) of each dose while naïve and control groups received 100 μL per day (s.c.) of physiological saline, after the onset of disease (day 28) for 13 days. On day 40, the mice were euthanized by cervical dislocation, lymph nodes were dissected, and limbs were surgically removed. Hind paws were processed for histologic analysis or homogenized for measurement of inflammatory mediators.

### 4.4. Arthritis Score

Joint inflammation was scored visually in each paw, while using a scale of 0–2, where 0-uninflamed, 1-mild, 1.5-marked, and 2–severe, as previously reported [54]. This macroscopic grading system assessed the extent of changes in redness, swelling, and ulceration of the paws. Scoring was performed every other day by two trained, independent observers (who were blinded with regard to experimental group), and weight measurements were also taken.

### 4.5. Histological Analysis

Ankles were kept in 4% paraformaldehyde in PBS (pH 7.4) for three weeks and decalcified with Osteosoft^®^ (Merck KGaA, Darmstadt, Germany) for four weeks. Ankles were dehydrated and then embedded in paraffin. Lateral sections of tissue (7 µm) were obtained while using a Leica microtome and mounted on SuperFrost slides (Menzel-Gläser/Thermofisher Scientific Inc., Waltham, MA, USA). Histopathological changes in joints were measured using a scoring system [54]. Haematoxylin and eosin staining was performed to study joint inflammation. The severity of inflammation in the joints was scored on a scale of 0–3 (0 = no cells, 1 = mild cellularity, 2 = moderate cellularity, and 3 = maximal cellularity). To study proteoglycan depletion from the cartilage matrix, sections were stained with safranin O, followed by counterstaining with fast green. The depletion of proteoglycan was determined using an arbitrary scale of 0–3, ranging from normal, fully stained cartilage to destained cartilage that was fully depleted of proteoglycan. Cartilage erosion was scored by assigning a value of 0–3, depending on the integrity of the joint cartilage. Chondrocyte death was determined by counting the number of gaps in the cartilage without any chondrocyte. TRAP staining was performed in order to determine osteoclasts by measuring the TRAP-positive area. Scoring was performed in a blinded manner by two independent observers. The scores are the result of the mean of three sections from each mouse. The sections were examined under a light microscope DM IL LED (Leica^®^, Wetzlar, Germany) and pictures were taken with a camera Leica^®^ (DFC 450 C).

### 4.6. Determination of Mediators in Paw Homogenates

Hind limbs were homogenized in liquid N_2_ with 2 mL of A buffer pH 7.4 (10 mM HEPES, pH 8, 1 mM EDTA, 1 mM EGTA, 10 mM KCl, 1 mM dithiothreitol, 5 mM NaF, 1 mM Na_3_VO_4_, 1 mg/mL leupeptin, 0.1 mg/mL aprotinine, and 0.5 mM phenylmethylsulfonyl fluoride). The tissue homogenates were sonicated (3 × 10 s) and centrifuged at 12,000× *g*, 10 min. at 4 °C. Supernatants were removed and used for determinations. TNFα, IL-1β, and IL-17 were measured by ELISA (R&D Systems, Minneapolis, MN, USA) (range of detection of 32–2700 pg/mL, 25–2000 pg/mL and 10.9-700 pg/mL, respectively). CXCL-1 was determined by ELISA (Promokine, Heidelberg, Germany) (8–1000 pg/mL). IL-2 and IL-10 were measured with Th1/Th2 Mouse Uncoated ELISA kit (Invitrogen, Thermofisher Scientific Inc.) (2–200 pg/mL and 30-4000 pg/mL, respectively). The IL-6 levels were determined by IL-6 mouse ELISA kit (Invitrogen, Thermofisher Scientific Inc.) (7.8–500 pg/mL). RANKL concentrations were assessed by TRANCE mouse ELISA kit (Invitrogen, Thermofisher Scientific Inc.) (2.74–2000 pg/mL). MPO activity, a neutrophil marker, was measured by a spectrophotometric method, as previously reported [55].

### 4.7. Serum Determinations

Blood was collected in heparinized tubes on day 40. After centrifugation at 12,000× *g*, serum was separated. DKK-1, sclerostin, and osteocalcin were determined by Multiplex assay while using the Merck-Millipore kit (assay range 15–60,000 pg/mL, 3–12,000 pg/mL and 146–600,000 pg/mL, respectively) (Merck KGaA). IgG2a was measured by IgG2a mouse ELISA kit (Thermofisher Scientific Inc.) (assay range 0.614–150 ng/mL).

### 4.8. Lymph Node Cells Isolation, Proliferation Assay and Cytokine Determination

Inguinal, popliteal, brachial, and axillary lymph nodes were dissected, mechanically disaggregated, and incubated in DMEM (Dulbecco’s Modified Eagle’s Medium) that was supplemented with fetal bovine serum (FBS) (10%), streptomycin/penicillin (1%), collagenase (1.6 mg/mL), and DNAse (200 μg/mL) (Merck KGaA) for 40 min. at 37 °C. After filtration with a 40 μm strainer, 1 mL of DMEM was added and the suspension was centrifuged at 500× *g*, 4 °C, 10 min. Medium was removed and the cells were resuspended in DMEM supplemented with FBS, streptomycin/penicillin and DNAse, as previously indicated. Cells were incubated for 15 min. at 37 °C. Subsequently, 500 μL of DMEM was added and the suspension was centrifuged. Medium was removed and cells were resuspended in PBS for flow cytometry or in DMEM that was supplemented with FBS (10%), streptomycin/penicillin (1%) for bromdeoxiuridine proliferation assay. Lymph node cells resuspended in DMEM were seeded into 96-well plates at 2.5 × 10 ^5^ cells/mL. At 24 h BrdU was added and the ELISA assay was performed at 48 h while using the cell proliferation ELISA BrdU from Merck KGaA. IL-2, IL-4, and IFNγ were measured by ELISA in the conditioned media of the cell proliferation assay with the Th1/Th2 Mouse Uncoated ELISA kit (Invitrogen, Thermofisher Scientific Inc.), with detection limits of 2–200 pg/mL, 4–500 pg/mL, and 15–2000 pg/mL, respectively.

### 4.9. Flow Cytometry

Cells that were obtained from lymph nodes were labeled with antibodies: antiCD3-Indo1(ref. 563565, lot 734861, clone 145-2C11), antiCD8-FITC (ref. 564422, lot 8037507, clone 53-6.7), antiRORγt-Pacific blue (ref. 562894, lot 8151861, clone Q31-378), and antiFoxP3-APC and antiCD4-PerCP-Cy5.5 (mouse Th17/Treg kit, ref. 51-9006647, lot 8067595) (BD Biosciences Europe, Madrid, Spain). Flow cytometry assays were performed with BD LSRFORTESSA (BD Biosciences). The software used was FACS DIVA 7.0 (BD Biosciences) and FlowJo v9 (BD Biosciences).

### 4.10. X-ray Analysis

Bone destruction in hind and front limbs was determined by X-ray analysis, which was carried out by microPET-CT (Albira) (Bruker, Billerica, MA, USA). A color scale represented bone density, where blue represents low density, yellow/green means middle density, and red high density. For numeric analysis, three-dimensional (3D) areas called region of interest (ROI) were used. These areas were exactly the same for all paws in 3 axis (X = 1.38 mm, Y = 2.94 mm, Z = 2.8 mm for hind limbs, and X = 2 mm, Y = 2 mm, and Z = 1 mm for front limbs) adjusted from the beginning of calcaneus, astragalus, and tarsus, until metatarsus for hind limbs and carpal bones for front limbs.

### 4.11. Statistical Analysis

Data are presented as mean ± S.D or S.E.M., with the number of individual values (*n*). Unless otherwise stated, duplicated determinations were done. Differences between experimental groups were tested by two-way ANOVA with Bonferroni post-test for the time course of arthritis macroscopic score and one-way ANOVA with Tukey’s post-test for all other data. Statistical analyses were performed with GraphPad PRISM 5.0 (GraphPad software, San Diego, CA, USA). A value of *p* < 0.05 was considered to be significant.
